# Development and validation of a risk nomogram for predicting recurrence in patients with non-valvular atrial fibrillation after radiofrequency catheter ablation

**DOI:** 10.1186/s12911-025-03338-4

**Published:** 2026-01-10

**Authors:** Yi Yu, Jin-Lan Chen, Guang-Yin Li, Shen-Shen Huang, Ting Wang, Xiao-Kai Li, Yi-Gang Li

**Affiliations:** 1 Department of Ultrasound, Shanghai Chest Hospital, Shanghai Jiao Tong University School of Medicine, No. 241 Huaihai Road, Shanghai, 200030 P.R. China; 2 Medical Imaging Technology, Shanghai University of Sport, No.399 Changhai Road, Yangpu District, Shanghai, 200438 P.R. China; 3 Department of Cardiology, Xinhua Hospital Affiliated to School of Medicine, Shanghai Jiaotong University, No.1665 Kongjiang Road, Shanghai, 200092 P.R. China

**Keywords:** Nomogram, Echocardiography, Systemic immune-inflammatory index, Non-valvular atrial fibrillation, Recurrence

## Abstract

**Background:**

Limited evidence exists regarding predictors of recurrence in patients with non-valvular atrial fibrillation (NVAF) following radiofrequency catheter ablation (RFCA). This study aimed to develop and validate a risk model for post-ablation recurrence in these patients.

**Methods:**

242 patients were enrolled and randomly divided into a modeling group (*n* = 169) and a validation group (*n* = 73) according to 7:3. The echocardiographic parameters, laboratory values and clinical features were used to derive a predictive model. Univariate and multivariate logistic regression analyses were used to identify independent risk factors.

**Results:**

During the 1-year follow-up, 87(36.00%) patients experienced AF recurrence. The nomogram was established by five variables including left atrial volume index (OR1.055, 95% CI: 1.021–1.090, *P* = 0.001), right atrial volume index (OR1.040, 95% CI: 1.008–1.073, *P* = 0.014), systemic immune-inflammatory index(OR1.015, 95% CI: 1.001–1.030, *P* = 0.036), New York Heart Association classification (OR 2.861, 95% CI: 1.282–6.383, *P* = 0.010), and CHA₂DS₂-VASc score(OR1.417, 95% CI: 1.077–1.864, *P* = 0.013). The model was developed, an online Dynamic Nomogram (https://cardiologyresearch.shinyapps.io/DynNomapp) application via the platform, and subsequently validated. The calculator achieved a C-index of 0.837(95% Cl: 0.774–0.899) in the training cohort and 0.895 (95% Cl: 0.823–0.968) in the validation cohort. The calibration curve showed good consistency between the predictions and observations in the training and validation cohorts. Decision curve analysis and clinical impact curves indicated the clinical utility of the nomogram.

**Conclusion:**

We developed a nomogram based on LAVI, RAVI, SII, NYHA classification, CHA₂DS₂-VASc score to estimate the risk of AF recurrence after RFCA. The newly developed nomogram demonstrated good discrimination and accuracy, suggesting its potential utility in predicting AF recurrence. Given its performance, the model is a promising tool; however, it is still in its preliminary stages and requires further validation with larger, multi-center, prospective studies involving diverse populations to confirm its generalizability.

**Supplementary information:**

The online version contains supplementary material available at 10.1186/s12911-025-03338-4.

## Introduction

Atrial fibrillation (AF) is one of the most common clinical arrhythmias, with its prevalence rising steadily due to population aging. Complications such as heart failure and embolism significantly increase patient disability and mortality rates, exacerbating the global healthcare burden [1].

Radiofrequency catheter ablation (RFCA), as a first-line rhythm control strategy for symptomatic AF, effectively improves patients’ quality of life [2, 3]. However, the 1-year post-procedural arrhythmia recurrence rate remains as high as 50%, and recurrent cases face a 2.3-fold increased risk of rehospitalization, severely limiting long-term clinical benefits [4, 5]. Predicting recurrence remains challenging, necessitating exploration of additional risk variables to aid clinical decision-making. Although numerous studies have investigated AF recurrence prediction models, the persistently high recurrence rate among the growing number of patients undergoing RFCA annually underscores unmet needs [6].

Echocardiography is the preferred modality for cardiac evaluation. Enhancing predictive capabilities through echocardiographic parameters for AF recurrence risk assessment remains clinically vital.

Inflammation plays an important role in AF, which can lead to atrial electrical remodeling and structural remodeling [7, 8]. However, few previous prediction models integrated with the inflammatory indicators to predict AF recurrence. The Systemic immune-inflammation index (SII) [9] was reported [10] to evaluate the intensity of systemic inflammatory status, and it might be useful for the prediction of AF recurrence.

The aim of our study was to develop and validate a nomogram for evaluating the risk of recurrence in NVAF patients after RFCA, and the model will be visualized with further development of an interactive web-based dynamic nomogram for clinical implementation, so that physicians can intervene in high-risk patients early and reduce the rate of AF recurrence after RFCA.

## Materials and methods

### Study population

This retrospective study enrolled patients with NVAF who underwent successful RFCA between September 2022 and November 2023, aged from 45 to 80 years, with a 12-month follow-up period. Based on the inclusion and exclusion criteria, 242 patients were eligible for analysis. Patients with any of the following conditions were excluded from study participation: (1) implantable cardioverter-defibrillator (ICD); (2) cardiomyopathy; (3) a history of tumor; (4) AF secondary to valve disease and post-valve replacement; (5) severe renal insufficiency requiring maintenance dialysis; (6) any missing data in the clinical information; (7) patients who were lost at follow-up after ablation; (8) incomplete clinical or echocardiographic data, lost to follow-up or had a follow-up time less than 3 months.

### Radiofrequency ablation operation

All patients underwent circumferential pulmonary vein isolation via RFCA [11]. Supplementary ablation (including left atrial roof line, posterior line, anterior line, or mitral isthmus ablation) was performed when indicated. Some patients also underwent direct current cardioversion if AF still existed after initial ablation. Post-procedurally, all patients received oral anticoagulant drugs for at least three months.

### Definition and follow up

AF classification into paroxysmal AF and non-paroxysmal AF (persistent, long-standing persistent, or permanent AF). NYHA functional classification categorized into four classes according to the 2022 AHA/ACC/HFSA Heart Failure Guidelines [12]. Patients were followed up regularly in our clinic at 3, 6 and 12 months post-procedure, with 12-lead ECG and 24-hour Holter monitoring performed at each visit. Those reporting AF-related symptoms (e.g., palpitations, chest pain, or fatigue) were advised to undergo additional ECG/Holter monitoring. After 6 months post-RFCA, follow-up continued either in-clinic or remotely by telephone.

AF recurrence was defined as any atrial tachyarrhythmias (AF, atrial flutter, and atrial tachycardia) that lasted over 30 seconds more than 3 months after the ablation [12]. The LAVI and RAVI were quantified from the apical 4-chamber and 2-chamber views using the biplane method of discs (modified Simpson’s rule). All volumetric measurements were manually traced at end-systole, defined as the frame preceding mitral valve opening. To ensure accuracy and consistency, all echocardiographic measurements were performed by a single, experienced echocardiographer who was blinded to the patient’s clinical outcomes. To guarantee consistency, the operator’s measurement reproducibility was tested and confirmed prior to the formal analysis using a set of pre-defined criteria, minimizing the potential for intra-observer variation. SII was calculated as follows: SII = [neutrophil count (×10^9^/L) × platelet count (×10^9^/L)]/lymphocyte count (×10^9^/L) [10, 13]. Severe renal disease was defined as estimated glomerular filtration rate (eGFR) < 30 mL/min·1.73 m^2^. Heart valve disease includes (mitral, tricuspid, aortic, pulmonary, etc.) and cardiomyopathy (hypertrophic cardiomyopathy and dilated cardiomyopathy, etc.)

### Data collection

Baseline and clinical characteristics, laboratory values and echocardiographic data were collected from the medical record system by trained physicians who were blinded to the aim of the study. Demographics and clinical characteristics were collected from patients including age, gender, body mass index (BMI), type of AF (paroxysmal vs. persistent), CHA₂DS₂-VASc score, medical history. 12-lead electrocardiogram (ECG), and 24-hour Holter were obtained for analysis. Echocardiographic parameters including left atrial volume index (LAVI), right atrial volume index (RAVI), et al. and the laboratory values were recorded including eGFR, N-terminal pro-B-type natriuretic peptide (NT-proBNP), neutrophil, lymphocyte and platelet counts, et al. For clarity of presentation in tables and figures, SII values are presented as divided by 10.

### Statistical analysis

All statistical analyses were performed using SPSS version 24.0 (SPSS Inc., Chicago, IL, USA) and the statistical package R, Version 4.3.3. Continuous variables were presented as mean ±standard deviation (Mean ± SD), while quantitative data with a skewed distribution are expressed as the median [M (Q25, Q75)]. Categorical variables were described as percentages (%). For normally distributed continuous variables, independent samples *t*-tests were employed for comparison; for non-normally distributed continuous variables, the Mann-Whitney U test was used for analysis. Comparisons of categorical variables were performed using the χ^2^ test or Fisher’s exact test, based on the data distribution.

Variables showing a univariate difference with *p* < 0.1 between the recurrence and non-recurrence groups were included in univariate logistic regression analysis for preliminary screening of risk factors associated with NVAF recurrence. Subsequently, variables with *p* < 0.1 in the univariate analysis were incorporated into multivariate logistic regression analysis using bidirectional stepwise regression (forward and backward stepwise regression) to identify independent predictors of AF recurrence.

Prior to performing multivariate regression analysis, the variance inflation factor (VIF) was calculated using the “rms” and “car” packages in R to assess multicollinearity among the predictor variables. Predictors with a VIF < 5 were considered free of significant multicollinearity and were retained for subsequent analysis. In the multivariate regression analysis, variables achieving a statistical significance threshold of *p* < 0.05 were selected and incorporated into the prediction model.

The nomogram was plotted using the “rms” package in R to visually represent the model’s predictive ability. This dynamic nomogram was converted into an interactive Shiny web application using the DNbuilder function from the “DynNom” package, facilitating real-time clinical utility for physicians.

The “pROC” package was employed to plot the receiver operating characteristic (ROC) curve. The diagnostic performance was assessed using the area under curve (AUC) or concordance index (C-index), interpreted as follows: low (0.5–0.7), moderate (0.7–0.9), or high ( > 0.9). Additionally, the model’s calibration was evaluated using the Hosmer-Lemeshow (H-L) test. A test *P*-value > 0.05 indicated good agreement between predicted and observed probabilities, signifying adequate fit. Calibration curves were also plotted using the calibrate function from the “rms” package to visually assess prediction accuracy. Finally, decision curve analysis (DCA) was performed using the “rmda” package to evaluate the model’s clinical utility and inform optimal treatment strategies.

## Results

### Patient characteristics

**Fig. 1:**
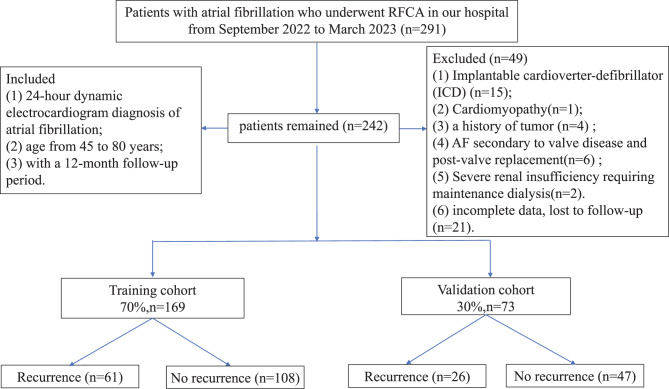
The study flow chart. RFCA: radiofrequency catheter ablation

A preliminary screening identified 291 patients with non-valvular atrial fibrillation (NVAF) who underwent radiofrequency catheter ablation (RFCA). After applying the exclusion criteria, 49 patients were excluded: 21 (7.20%) for missing data and 28 for other reasons. Consequently, a final cohort of 242 patients was enrolled in the study. These patients were randomly allocated 7:3 to a modeling group (*n* = 169) and a validation group (*n* = 73). The study flow chart is presented in Fig. 1. Baseline characteristics of 242 patients are summarized in Table 1. Baseline characteristics of the 21 patients excluded due to missing data are presented in the supplementary file 1. We compared the baseline characteristics (e.g., age, sex, key clinical outcomes) between these two groups. This analysis revealed no statistically significant differences in any of the key baseline variables or the primary outcome between the two groups.

**Table 1:** Baseline characteristics of the patients with non-valvular atrial fibrillation

Variables	No. (%) Total (*N* = 242)	No-recurrence group (*n* = 155)	Recurrence group (*n* = 87)	*P-* value
Age, $\bar \chi$±s, y	69.10 ± 8.50	68.20 ± 8.70	70.70 ± 7.90	0.026
Gender, n (%)				0.193
Male	134 (55.40)	81 (52.30)	53 (60.90)	
Female	108 (44.60)	74 (47.70)	34 (39.10)	
CHA_2_DS_2_-VASc Score, $\bar \chi$±*s*	3.30 ± 1.50	3.00 ± 1.40	3.90 ± 1.50	< 0.001
Type of AF, n (%)				0.843
Paroxysmal AF	130 (53.70)	84 (54.20)	46 (52.90)	
Non- paroxysmal AF	112 (46.30)	71 (45.80)	41 (47.10)	
Hypertension, n (%)				0.034
Yes	172 (71.10)	103 (66.50)	69 (79.30)	
No	70 (28.90)	52 (33.50)	18 (20.69)	
Diabetes, n (%)				0.323
Yes	58 (24.00)	34(21.90)	24 (27.60)	
No	184 (76.00)	121 (78.10)	63 (72.40)	
Stroke, n (%)				0.389
Yes	43 (17.80)	30 (19.40)	13 (14.90)	
No	199 (82.20)	125 (80.60)	74 (85.10)	
Smoking, n (%)				0.362
Yes	56 (23.10)	33 (21.30)	23 (26.40)	
No	186 (76.90)	122 (78.70)	64 (73.60)	
CAD, n (%)				0.163
Yes	133 (55.00)	80 (51.60)	53 (60.90)	
No	109 (45.00)	75 (48.40)	34 (39.10)	
NYHA classification, n (%)				< 0.001
Class I-II	135 (55.80)	105 (67.70)	30 (34.50)	
Class III-IV	107 (44.20)	50 (32.30)	57 (65.50)	
Echocardiographic parameters				
LAVI, ml/m^2^	48 ± 18	42 ± 10	59± 24	< 0.001
RAVI, ml/m^2^	39 ± 16	35± 12	47 ± 19	< 0.001
LVDD, mm	48.30 ± 5.20	47.70± 5.10	49.20 ± 5.20	0.036
LVDS, mm	31.40± 5.40	30.90 ± 5.20	32.20 ± 5.70	0.060
LAD, mm	42.00(38.00,45.20)	41.70(38.00,44.00)	43.60(39.20,47.00)	0.007
LVEF, %	62.50 ± 8.00	63.20 ± 7.50	61.20 ± 8.50	0.063
LVEDV, ml	110.50 ± 26.80	107.80± 6.30	115.40± 27.00	0.033
LVESV, ml	40.40 ± 18.00	38.30± 17.10	44.10 ± 19.00	0.014
HR, bpm	76± 20	76± 21	76 ± 20	0.998
CO, L/min	5.13 ± 1.85	5.09 ± 1.79	5.20 ± 1.95	0.661
MRVmax, m/s	3.76 ± 1.11	3.78 ± 1.14	3.72 ± 1.08	0.682
TRVmax, m/s	2.63 ± 0.47	2.67 ± 0.46	2.57 ± 0.49	0.133
TRSP, mmHg	36.30 ± 8.10	35.60 ± 7.70	37.40 ± 8.70	0.090
Laboratory values				
eGFR, mL/min/1.73 m^2^	75.21 ± 27.12	76.00± 27.25	73.81 ± 26.99	0.546
CRP, mg/L	0.90 (0.90, 2.48)	0.90(0.90,2.00)	1.00 (0.90, 3.00)	0.744
CK-MB, ng/mL	1.35(0.78, 2.80)	1.30(0.68, 2.73)	1.50(0.80, 3.50)	0.239
cTnI, ng/mL	0.008 (0.006, 0.013)	0.009(0.006, 0.013)	0.007 (0.005, 0.013)	0.546
NT-proBNP(pg/mL)	662.16 (193.60, 1122.81)	542.90(165.20,1071.10)	798.50 (328.50,1277.80)	0.093
D-dimer, mg/L FEU	0.27(0.19, 0.44)	0.28 (0.19, 0.43)	0.25 (0.19, 0.55)	0.659
White blood cell, $\times$10^9^/L	5.90(4.91, 7.09)	6.02(5.03, 7.08)	5.51 (4.75, 7.08)	0.333
Neutrophils, $\times$10^9^/L	3.52 (2.87, 4.47)	3.52 (2.89, 4.49)	3.52(2.79, 4.44)	0.990
Monocyte, $\times$10^9^/L	0.41 (0.32, 0.52)	0.41 (0.32, 0.53)	0.41(0.34,0.51)	0.850
Lymphocyte, $\times$10^9^/L	1.60 (1.28, 2.04)	1.61(1.39, 2.08)	1.54(1.16, 1.97)	0.046
Erythrocyte,$\times$10^12^/L	4.45 (3.99, 4.79)	4.46 (4.00, 4.80)	4.40 (3.98, 4.78)	0.928
Platelet, $\times$10^9^/L	153.00 (128.00, 178.76)	142.00(118.50,167.32)	173.19(150.31,208.43)	< 0.001
SII	32.60 (23.00, 48.20)	28.00 (20.20, 39.20)	42.50 (28.90, 61.60)	< 0.001

AF: atrial fibrillation; BMI: body mass index; CAD: coronary heart disease; NYHA: New York Heart Association; LAVI: left atrial volume index; RAVI: right atrial volume index; LVDD: left ventricular diastolic diameter; LVDS: left ventricular systolic diameter; LAD: left atrial diameter; LVEF: left ventricular ejection fraction; LVEDV: left ventricular end-diastolic volume; LVESV: left ventricular end-systolic volume; HR: heart rate; CO: cardiac output; MRVmax: mitral regurgitation velocity (max); TRVmax: tricuspid regurgitation velocity (max); TRSP: tricuspid regurgitation systolic pressure; eGFR: estimated glomerular filtration rate; CRP: c-reactive protein; CK-MB: creatine kinase-mb; cTnI: cardiac troponin I;NT-proBNP: N-terminal pro-brain natriuretic peptide; SII: systemic immune-inflammation index (values presented as SII/10)

Continuous variables are generally reported to two decimal places (e.g., mean ± SD), except for specific clinical indices (e.g., LAVI, RAVI, HR, et al.) which are reported as whole numbers and cTnI which is reported to three decimal places

1. The 242 NVAF patients had a mean age of 69.10 ± 8.50 years, 134 (55.40%) patients were male and 108 (44.60%) were female. Within 1-year post-RFCA, AF recurrence occurred in 87 patients (87/242, 36.00%). Recurrence rates were comparable between the modeling group (61/169, 36.10%) and validation group (26/73, 35.60%).
2. Compared to the non-recurrence group, patients in the recurrence group had higher rates of hypertension, greater proportions in NYHA functional class III-IV, increased age, elevated CHA₂DS₂-VASc scores, larger LAVI, larger RAVI, larger LVDD, LAD, LVEDV and LVESV, platelet count, and SII were also elevated (*p* < 0.05 for all).
3. Between the modeling and the validation groups, significant differences were observed only in AF type and cTnI levels (*p* < 0.05). No statistically significant differences were found in any other variables (Table 2).

**Table 2:** Comparison of the information in the training and validation cohorts

Variables	Training cohort *n* = (169)	Validation cohort (*n* = 73)	*p*- value
Age, $\bar \chi$±s, y	68.80 ± 8.10	69.90 ± 9.20	0.323
Gender, n (%)			0.871
Male	93 (55.00)	41 (56.20)	
Female	76 (45.00)	32 (43.80)	
BMI, kg/m^2^	25.18 ± 3.78	24.84 ± 3.19	0.500
CHA_2_DS_2_-VASc Score, $\bar \chi$±*s*	3.40 ± 1.50	3.10 ± 1.60	0.185
Type of AF, n (%)			0.043
Paroxysmal AF	98 (58.00)	32 (43.80)	
Non- paroxysmal AF	71 (42.00)	41 (56.20)	
Hypertension, n (%)			0.373
Yes	123 (72.80)	49 (67.10)	
No	46 (27.20)	24 (32.90)	
Diabetes, n (%)			0.140
Yes	45 (26.60)	13 (17.80)	
No	124 (73.40)	60 (82.20)	
Stroke, n (%)			0.722
Yes	31 (18.30)	12 (16.40)	
No	138 (81.70)	61 (83.60)	
Smoking, n (%)			0.484
Yes	37 (21.90)	19 (26.00)	
No	132 (78.10)	54 (74.00)	
CAD, n (%)			0.150
Yes	98 (57.99)	35 (47.95)	
No	71 (42.01)	38 (52.06)	
NYHA classification, n (%)			0.838
Class I-II	95 (56.20)	40 (54.80)	
Class III-IV	74 (43.80)	33 (45.20)	
Echocardiographic parameters			
LAVI, ml/m^2^	47 ± 19	50 ± 15	0.229
RAVI, ml/m^2^	38 ± 15	41 ± 17	0.195
LVDD, mm	48.20 ± 5.10	48.40 ± 5.30	0.709
LVDS, mm	31.10 ± 5.20	31.90 ± 5.90	0.347
LAD, mm	42.00(38.00, 45.00)	42.00(39.00, 45.60)	0.554
LVEF, %	62.90 ± 7.70	61.50 ± 8.30	0.213
LVEDV, ml	110.20 ± 26.90	111.30 ± 26.60	0.784
LVESV, ml	39.10 ± 16.70	43.30 ± 20.40	0.095
HR, bpm	76 ± 20	75 ± 20	0.775
CO, L/min	5.26 ± 1.89	4.84 ± 1.72	0.100
MRVmax, m/s	3.73 ± 1.19	3.82 ± 0.95	0.563
TRVmax, m/s	2.60 ± 0.50	2.70 ± 0.39	0.160
TRSP, mmHg	35.90 ± 8.63	37.01 ± 6.82	0.328
Laboratory values			
eGFR, mL/min/1.73 m^2^	75.39 ± 26.90	74.80 ± 27.81	0.877
CRP, mg/L	0.90(0.90,3.00)	1.00 (0.90, 2.00)	0.825
CK-MB, ng/mL	1.40(0.70, 2.80)	1.30(0.80, 2.93)	0.931
cTnI, ng/mL	0.007(0.005,0.011)	0.011(0.007,0.014)	0.025
NT-proBNP(pg/mL)	659.10(191.60,1185.50)	679.70(197.20,964.80)	0.801
D-dimer, mg/L FEU	0.28(0.19, 0.45)	0.23(0.19,0.42)	0.158
White blood cell, $\times$10^9^/L	5.98(4.95,7.15)	5.83(4.82, 6.93)	0.485
Neutrophils, $\times$10^9^/L	3.57(2.97,4.54)	3.27(2.66, 4.18)	0.170
Monocyte, $\times$10^9^/L	0.40(0.32,0.52)	0.42(0.32, 0.50)	0.911
Lymphocyte, $\times$10^9^/L	1.56(1.25,2.04)	1.68(1.37, 2.04)	0.334
Erythrocyte,$\times$10^12^/L	4.46(4.00, 4.81)	4.44(3.95, 4.76)	0.943
Platelet, $\times$10^9^/L	153.00(128.00,179.00)	153.0(128.00,178.00)	0.975
SII	33.50(23.60,50.70)	28.90(20.50,43.60)	0.153

Within the modeling group, compared to the non-recurrence group, patients in the recurrence group had higher proportions in NYHA functional class III-IV, higher CHA₂DS₂-VASc scores, larger LAVI, larger RAVI, and elevated white blood cell count, lymphocyte count, platelet count, and SII (*p* < 0.05 for all), (Table 3).

**Table 3:** Comparison of the information in No-recurrence and recurrence groups of the training cohort

Variables	No-recurrence group (*n* = 108)	Recurrence group (*n* = 61)	*P*- value
Age, $\bar \chi$±s, y	68.30 ± 8.30	69.50 ± 7.90	0.366
Gender, n (%)			0.434
Male	57 (52.80)	36 (59.00)	
Female	51 (47.20)	25 (41.00)	
BMI, kg/m^2^	24.83 ± 3.42	25.81± 4.30	0.103
CHA_2_DS_2_-VASc Score, $\bar \chi$±*s*	3.10 ± 1.40	3.92 ± 1.45	< 0.001
Type of AF, n (%)			0.597
Paroxysmal AF	61 (56.50)	37 (60.70)	
Non- paroxysmal AF	47 (43.50)	24 (39.30)	
Hypertension, n (%)			0.098
Yes	74 (68.50)	49 (80.30)	
No	34 (31.50)	12 (19.70)	
Diabetes, n (%)			0.784
Yes	28 (25.90)	17 (27.80)	
No	80 (74.10)	44 (72.10)	
Stroke, n (%)			0.938
Yes	20 (18.50)	11 (18.00)	
No	88 (81.50)	50 (82.00)	
Smoking, n (%)			0.306
Yes	21 (19.4)	16 (26.20)	
No	87 (80.6)	45 (73.80)	
CAD, n (%)			0.394
Yes	60 (55.60)	38 (62.30)	
No	48 (44.40)	23 (37.70)	
NYHA classification, n (%)			0.003
Class I-II	70 (64.80)	25 (41.00)	
Class III-IV	38 (35.20)	36 (59.00)	
Echocardiographic parameters			
LAVI, ml/m^2^	41 ± 11	58 ± 6	< 0.001
RAVI, ml/m^2^	35 ± 12	45 ± 18	< 0.001
LVDD, mm	47.60± 5.30	49.10 ± 4.80	0.079
LVDS, mm	30.90 ± 5.40	31.60 ± 4.90	0.362
LAD, mm	41.50± 5.80	43.30 ± 7.00	0.064
LVEF, %	63.00 ± 7.90	62.80 ± 7.60	0.864
LVEDV, ml	107.70 ± 27.00	114.70 ± 26.40	0.107
LVESV, ml	38.00 ± 17.60	41.00 ± 15.00	0.271
HR, bpm	77 ± 21	75±20	0.659
CO, L/min	5.20 ± 1.90	5.37 ± 1.89	0.581
MRVmax, m/s	3.77 ± 1.25	3.67 ± 1.07	0.596
TRVmax, m/s	2.65 ± 0.48	2.52 ± 0.52	0.114
TRSP, mmHg	35.53 ± 8.36	36.54 ± 9.12	0.467
Laboratory values			
eGFR, mL/min/1.73 m^2^	76.43 ± 27.58	73.54 ± 25.77	0.504
CRP, mg/L	0.90(0.90, 2.00)	0.90(0.90, 4.00)	0.627
CK-MB, ng/mL	1.20(0.60,2.60)	1.60(0.90, 4.40)	0.052
cTnI, ng/mL	0.007(0.006, 0.011)	0.007(0.005, 0.011)	0.593
NT-proBNP(pg/mL)	562.10(182.20,1125.50)	761.90(322.80, 1218.50)	0.413
D-dimer, mg/L FEU	0.30(0.19, 0.44)	0.26(0.19, 0.59)	0.938
White blood cell, $\times$10^9^/L	6.26(5.19,7.26)	5.26 (4.69, 6.84)	0.028
Neutrophils, $\times$10^9^/L	3.63(3.01,4.77)	3.52(2.73, 4.26)	0.324
Monocyte, $\times$10^9^/L	0.41(0.33,0.53)	0.38(0.32, 0.51)	0.217
Lymphocyte, $\times$10^9^/L	1.59(1.38,2.09)	1.46(1.14, 1.91)	0.030
Erythrocyte,$\times$10^12^/L	4.47 (4.07, 4.83)	4.27 (3.98, 4.78)	0.353
Platelet, $\times$10^9^/L	143.32(118.75,167.00)	173.19(147.12,209.00)	< 0.001
SII	30.60(21.80, 40.00)	42.40 (31.40, 64.40)	< 0.001

### Predictors of AF recurrence in univariate and multivariate analysis

Univariate logistic regression identified the following significant predictors of AF recurrence (*p* < 0.05) (Table 4). NYHA functional classification: OR 2.653 (95% CI: 1.391–5.057), *p* = 0.003; LAVI: OR 1.075 (95% CI: 1.043–1.107), *p* < 0.001; RAVI: OR 1.054 (95% CI: 1.028–1.081), *p* < 0.001; SII: OR 1.022 (95% CI: 1.009–1.036), *p* = 0.001; CHA₂DS₂-VASc score: OR 1.486 (95% CI: 1.179–1.872), *p* < 0.001.

**Table 4:** Univariate logistic regression analysis of recurrence based on data in the training cohort

Variables	β	S.E.	Z	OR (95%CI)	*P*- value
NYHA classification, n (%)					
Class I-II				1.000（reference）	
Class III-IV	0.976	0.329	2.963	2.653 (1.391 ~ 5.057)	0.003
LAVI, ml/m^2^	0.072	0.015	4.766	1.075 (1.043 ~ 1.107)	< 0.001
RAVI, ml/m^2^	0.053	0.013	4.057	1.054 (1.028 ~ 1.081)	< 0.001
SII	0.022	0.007	3.225	1.022 (1.009 ~ 1.036)	0.001
CHA_2_DS_2_-VASc Score, $\bar \chi$±*s*	0.396	0.118	3.357	1.486 (1.179 ~ 1.872)	< 0.001
Hypertension n (%)					
Yes	0.629	0.383	1.643	1.876 (0.886 ~ 3.974)	0.100
No				1.000 （reference）	
LVDD, mm	0.056	0.032	1.743	1.057 (0.993 ~ 1.126)	0.081
LAD, mm	0.047	0.026	1.820	1.049 (0.996 ~ 1.103)	0.069
White blood cell, $\times$10^9^/L	−0.171	0.099	−1.728	0.843 (0.695 ~ 1.023)	0.084
Lymphocyte, $\times$10^9^/L	−0.468	0.290	−1.618	0.626 (0.353 ~ 1.112)	0.106
Monocyte, $\times$10^9^/L	−1.418	1.092	−1.299	0.242 (0.028 ~ 2.059)	0.194
Platelet, $\times$10^9^/L	0.028	0.006	5.058	1.029 (1.017 ~ 1.040)	< 0.001

OR: odds ratio; Cl: confidence interval

Multivariate logistic regression identified the following five independent predictors of AF recurrence after ablation (Table 5): NYHA functional classification: OR 2.861 (95% CI: 1.282–6.383), *p* = 0.010; LAVI: OR 1.055 (95% CI: 1.021–1.090), *p* = 0.001; RAVI: OR 1.040 (95% CI: 1.008–1.073), *p* = 0.014; SII: OR 1.015 (95% CI: 1.001–1.030), *p* = 0.036;CHA₂DS₂-VASc score: OR 1.417 (95% CI: 1.077–1.864), *p* = 0.013.

**Table 5:** Multivariate logistic regression analysis of recurrence based on data in the training cohort

Variables	β	S.E	Z	*P*- value	OR (95%CI)
Intercept	−10.229	2.109	−4.849	< 0.001	0.000 (0.000 ~ 0.002)
NYHA classification, n (%)					
Class I-II	/	/	/	/	（reference）
Class III-IV	1.051	0.410	2.567	0.010	2.861 (1.282 ~ 6.383)
LAVI, ml/m^2^	0.054	0.017	3.179	0.001	1.055 (1.021 ~ 1.090)
RAVI, ml/m^2^	0.039	0.016	2.452	0.014	1.040 (1.008 ~ 1.073)
SII	0.015	0.007	2.094	0.036	1.015 (1.001 ~ 1.030)
CHA_2_DS_2_-VASc Score, $\bar \chi$±*s*	0.348	0.140	2.487	0.013	1.417 (1.077 ~ 1.864)

### Predictive value of risk factors for recurrence after RFCA in NVAF patients

ROC analysis demonstrated significant predictive value (all *p* < 0.05) for the LAVI, RAVI, SII, CHA₂DS₂-VASc score, and NYHA functional classification regarding 1-year AF recurrence after RFCA: [LAVI: AUC 0.734(95% CI: 0.654–0.813), cut-off value 48, *p* < 0.001; RAVI: AUC 0.677(95% CI:0.596–0.759), cut-off value 31, *p* < 0.001; SII: AUC 0.683(95% CI:0.599–0.767), cut-off value 38.48, *p* < 0.001; CHA₂DS₂-VASc score: AUC 0.669(95% CI:0.583–0.756), cut-off value 4.50, *p* < 0.001; NYHA functional classification: AUC 0.619(95% CI: 0.531–0.708) *p* = 0.001] (Table 6).

**Table 6:** Predictive value of risk factors for recurrence after RFCA in NVAF patients

Variables	cut-off value	sensitivity	specificity	AUC (95%Cl)	*P*- value
LAVI, ml/m^2^	48	0.656	0.741	0.734 (0.654–0.813)	< 0.001
RAVI, ml/m^2^	31	0.886	0.417	0.677(0.596–0.759)	< 0.001
SII	38.48	0.574	0.731	0.683(0.599–0.767)	< 0.001
CHA_2_DS_2_-VASc Score, $\bar \chi$±*s*	4.50	0.852	0.311	0.669(0.583–0.756)	< 0.001
NYHA classification	——	0.590	0.648	0.619(0.531–0.708)	0.010

RFCA: catheter radiofrequency ablation; NVAF: non-valvular atrial fibrillation

### Model development and presentation

(1) Model development: collinearity diagnostics for the five predictors (NYHA functional classification, LAVI, RAVI, SII, and CHA₂DS₂-VASc score) revealed variance inflation factors (VIF) of 1.031, 1.518, 1.460, 1.060, and 1.066, respectively. All VIF values were < 5, indicating no significant multicollinearity among the predictors.

Using the multivariate regression coefficients, we developed the following logistic regression model:

logit(p) = −7.050 + 1.051 × NYHA functional classification + 0.015 × SII + 0.054 × LAVI + 0.039 × RAVI + 0.348 × CHA₂DS₂-VASc score

This model demonstrated the discriminative ability for 1-year recurrence risk, with an AUC of 0.837 (95% CI: 0.774–0.899).

**Fig. 2:**
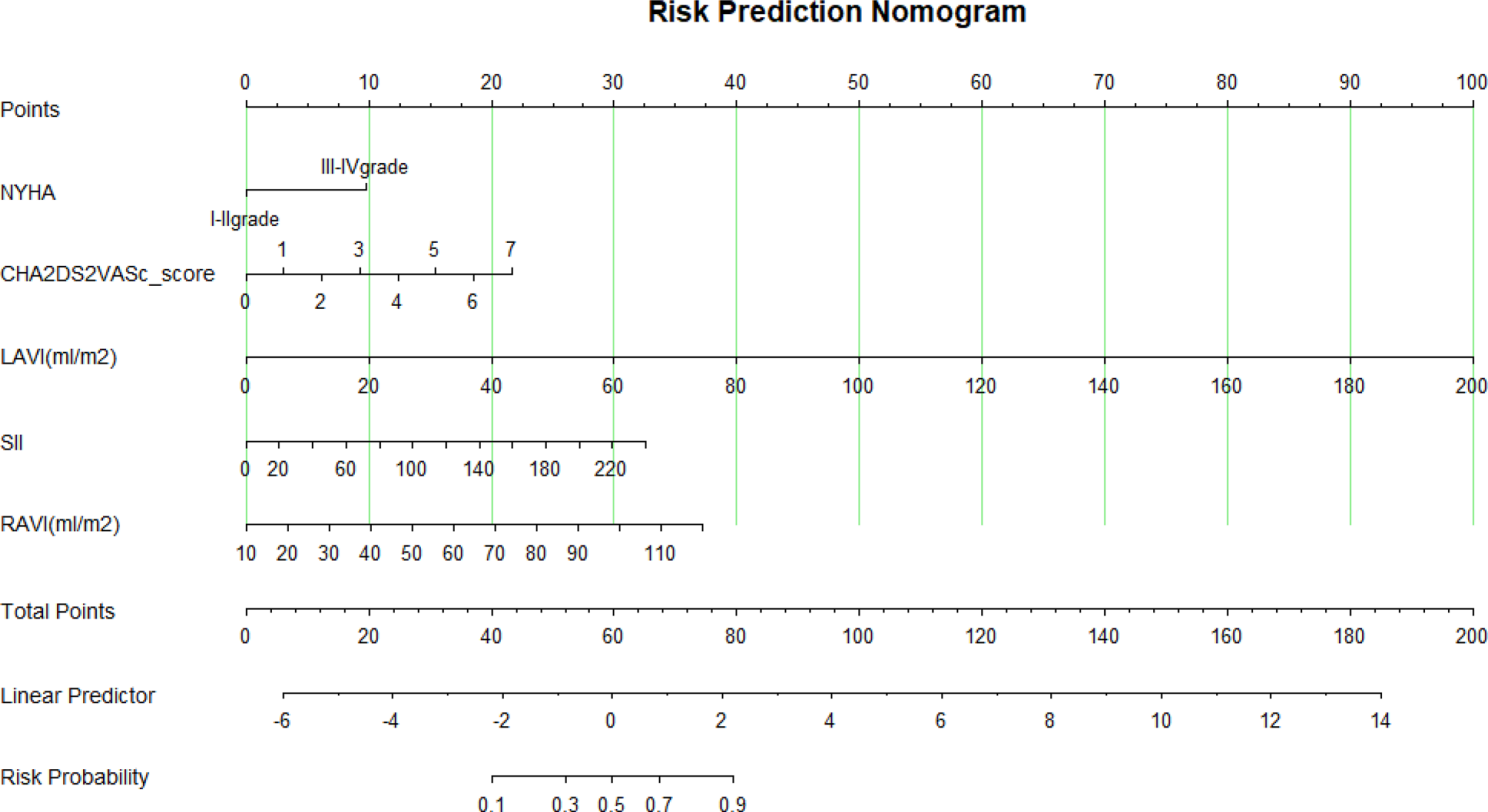
Nomogram for predicting recurrence after RFCA in NVAF patients. NYHA: New York Heart Association; LAVI: left atrial volume index; SII: systemic immune-inflammation index (values presented as SII/10); RAVI: right atrial volume index

(2) Nomogram presentation: A nomogram was constructed to predict the 1-year risk of AF recurrence after RFCA, incorporating the five independent predictors: NYHA functional classification, LAVI, RAVI, SII, and CHA₂DS₂-VASc score (Fig. 2).

### Clinical application of the web-based dynamic nomogram

**Fig. 3:**
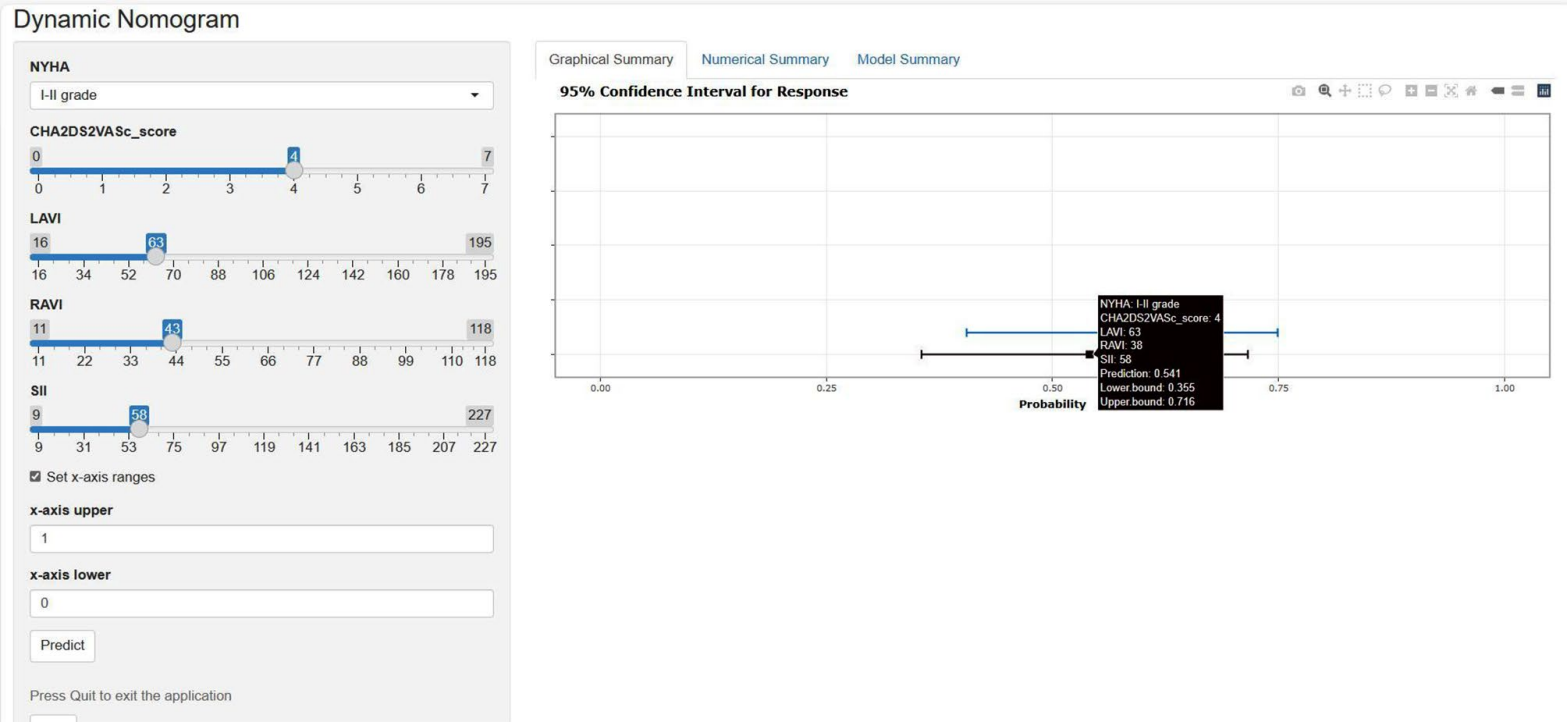
For a NVAF patient with the following pre-ablation characteristics: NYHA class: I-II, CHA₂DS₂-VASc score: 4.00, LAVI: 63 ml/m^2^, RAVI: 38 ml/m^2^, SII: 58.00 (values presented as SII/10). The dynamic nomogram predicted a 0.541 (95% ci: 0.405–0.749) probability of 1-year af recurrence. The link is https://cardiologyresearch.Shinyapps.io/DynNomapp

We developed a dynamic web-based nomogram application using the DynNom package and RStudio tools, hosted on the shinyapps.io platform. The application interface consists of two sections: Model controls (for variable adjustment) and plot display (for visualization),freely available at https://cardiologyresearch.shinyapps.io/DynNomapp, which allows the user to input predictor values to obtain a probability output of 1-year AF recurrence, as well as the associated risk class, for a specific patient (Fig. 3). For a NVAF patient with the following pre-ablation characteristics: NYHA class: I-II, CHA₂DS₂-VASc score: 4, LAVI: 63 ml/m^2^, RAVI: 38 ml/m^2^, SII: 58.00 (values presented as SII/10). The dynamic nomogram predicted a 0.541 (95% CI: 0.405–0.749) probability of 1-year AF recurrence.

The development and reporting of this prediction model followed the guidelines outlined in the Transparent Reporting of a multivariable prediction model for Individual Prognosis Or Diagnosis (TRIPOD) statement. The completed TRIPOD checklist is provided in the supplementary file 2.

### Evaluation and validation of the nomogram using ROC curves

**Fig. 4:**
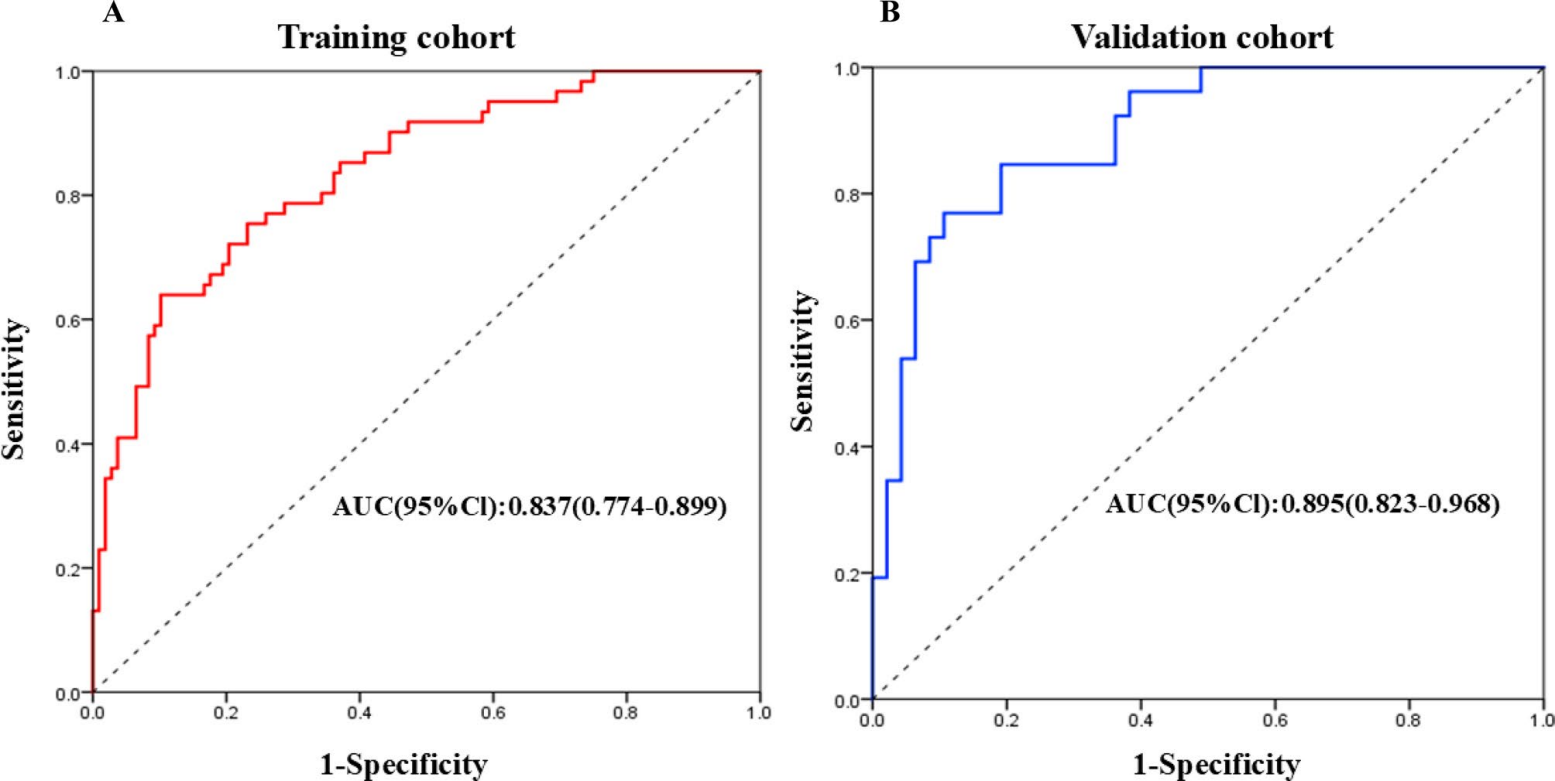
Evaluation and validation of the nomogram using ROC curves. (**A**) ROC curve in the training cohort, the nomogram demonstrated an AUC of 0.837 (95% ci: 0.774–0.899) with 89.80% sensitivity and 63.90% specificity for predicting af recurrence. (**B**) ROC curve in the validation cohort, the nomogram identified an AUC of 0.895 (95% ci: 0.823–0.968) with 89.40% sensitivity and 76.90% specificity for predicting af recurrence. ROC: receiver operating characteristic; CI: confidence interval

The nomogram demonstrated the predictive value with a C-index of 0.837 (95% CI: 0.774–0.899) in the training cohort and 0.895 (95% CI: 0.823–0.968) in the validation cohort (Fig. 4).

To assess the robustness of our final model which excluded AF type, we performed a sensitivity analysis by incorporating the “AF type” variable. The resulting model demonstrated a nearly identical discriminative performance (C-statistic = 0.823) compared to the original model (C-statistic = 0.820).

### Performance of the model in the validation cohort

The model exhibited high discriminative performance in the validation cohort with a C-index of 0.895 (95% CI: 0.823–0.968). The result of Hosmer-Lemeshow (H-L) testing showed χ^2^ = 4.86, *p* = 0.955 in the training cohort and χ^2^ = 11.85, *p* = 0.158 in the validation cohort, which was indicative of good calibration. The *p*-values for both cohorts were greater than 0.05, meaning the null hypothesis could not be rejected. Therefore, the model demonstrated a good fit in the training and validation cohorts. As *p*-values exceeded 0.05 in both cohorts, the model demonstrated appropriate calibration accuracy.

**Fig. 5:**
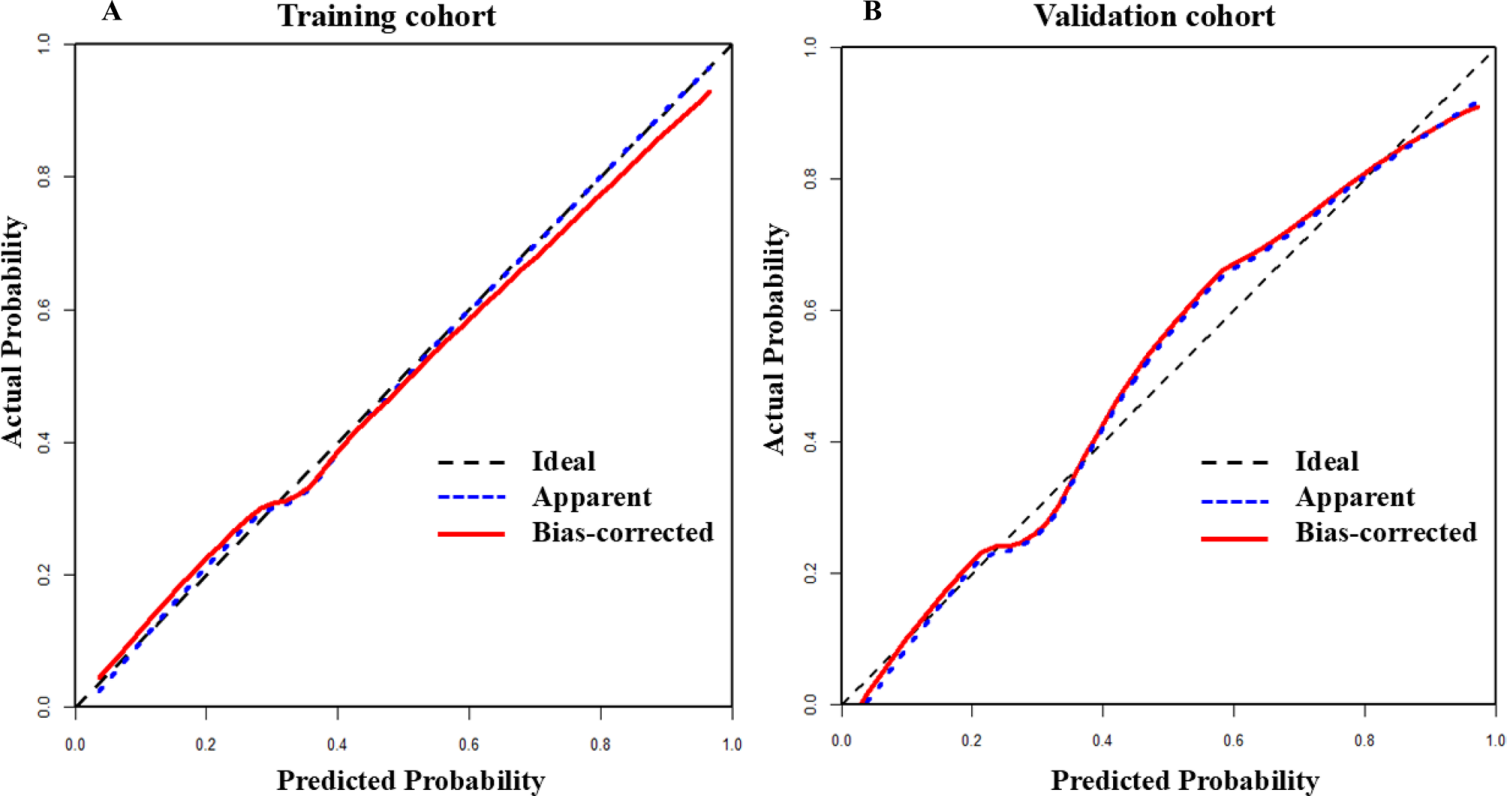
Evaluation and validation of the nomogram calibration curve. (**A**) calibration curve in the training cohort; (**B**) calibration curve in the validation cohort. The x-axis represents the overall predicted probability of af recurrence after RFCA, and the y-axis represents the actual probability. The model calibration is indicated by the degree of fit of the curve and the diagonal line. The Hosmer-Lemeshow (H-L) test evaluated model calibration, where failure to reject the null hypothesis (*p* > 0.05) indicates adequate fit. The H-L test results showed χ^2^ = 4.86, *p* = 0.955 in the training cohort and χ^2^ = 11.85, *p* = 0.158 in the validation cohort. The p-values for both cohorts were greater than 0.05, meaning the null hypothesis could not be rejected. Therefore, the model demonstrated a good fit in both the training and validation cohorts. As *p*-values exceeded 0.05 in both cohorts, the model demonstrated appropriate calibration accuracy

A bias-corrected calibration curve was constructed using 1000 Bootstrap resamples. This revealed that the recurrence outcomes after RFCA predicted by the nomogram for NVAF patients aligned well with the actual observed outcomes (Fig. 5).

### Evaluation and validation of the model using DCA

**Fig. 6:**
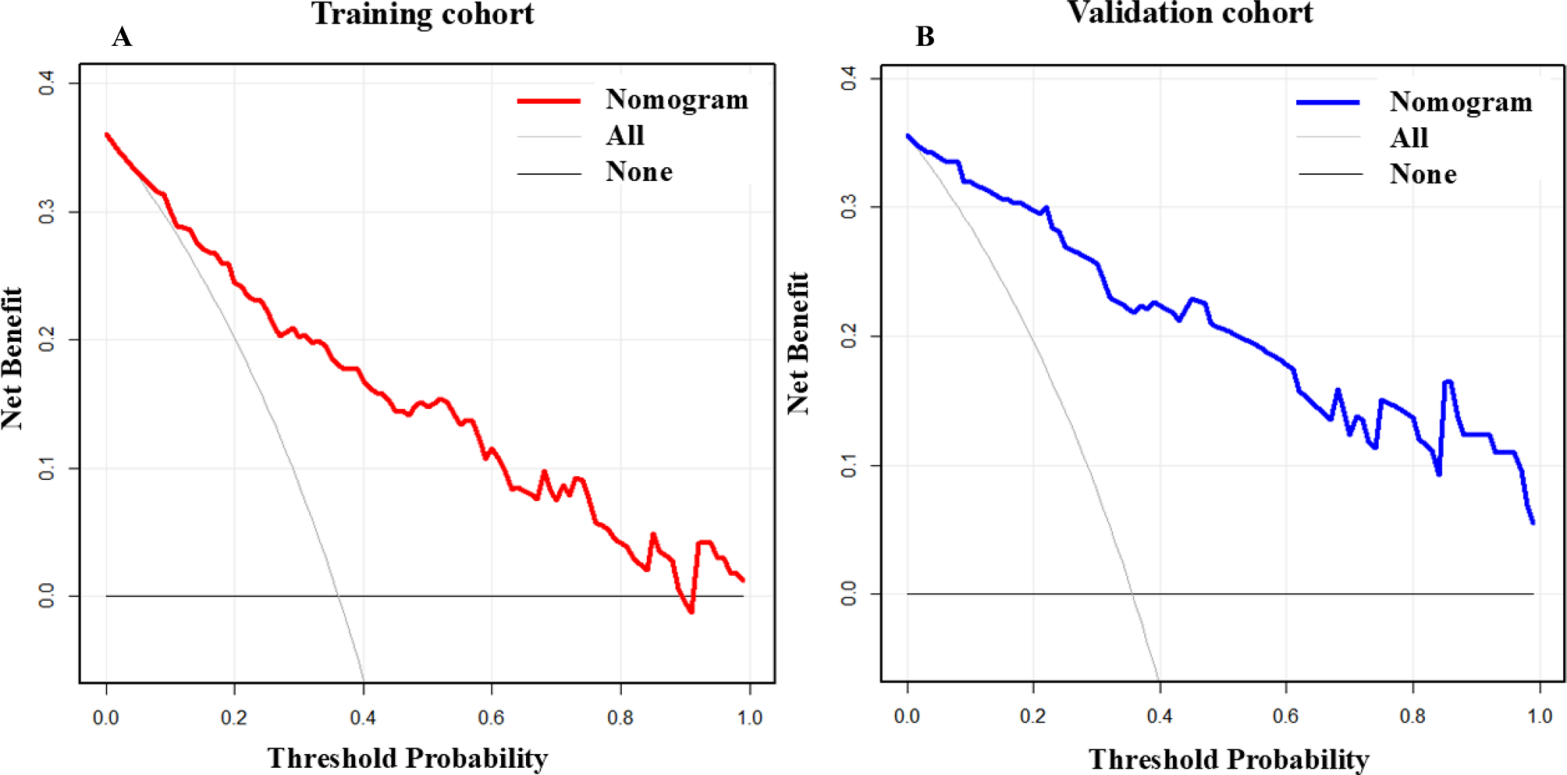
Decision curve analysis (DCA) of the prediction model. Decision curve analysis for the training cohort (**A**) and the validation cohort (**B**). The solid black line indicates that all samples are negative (no recurrence), implying no intervention was needed, with a net benefit of zero. The solid grey line indicates that all samples are positive (recurrence), implying all receive intervention. The solid red and blue lines represented the net benefit predicted by the nomogram. As shown in the figure, both the red and blue lines lied above and to the right of both the black and grey reference lines. This demonstrated that the nomogram provided positive clinical utility and could support clinical decision-making

The DCA incorporated two reference strategies: the solid black line represented the “treat none” strategy (all samples classified as negative for recurrence, implying no intervention), yielding a net benefit of zero. The solid grey line represented the “treat all” strategy (all samples classified as positive for recurrence, implying universal intervention). The nomogram’s predictive performance was shown by the solid red line (training cohort) and blue line (validation cohort). As illustrated, both the training and validation cohort curves lied above and to the right of the “treat none” and “treat all” reference lines across clinically relevant threshold probabilities. This demonstrated that the nomogram provided positive net clinical utility and could effectively support clinical decision-making (Fig. 6).

The DCA also demonstrated that the nomogram provides a net benefit over a wide range of threshold probabilities, approximately from 20% to 60%. This range represents a plausible spectrum of clinician and patient preference regarding the decision to initiate more intensive post-ablation management. For instance, a threshold of 20% reflects a higher willingness to intervene to avoid a missed recurrence, whereas a threshold of 60% reflects a much higher aversion to unnecessary treatment.

## Discussion

In this study, we found LAVI, RAVI, SII, NYHA class III-IV and CHA₂DS₂-VASc score were the independent predictors significantly associated with AF recurrence after RFCA within 1-year follow-up. A recurrence risk prediction model was developed based on these parameters and visually presented as a nomogram. Furthermore, this study implemented a web-based dynamic nomogram tool that provides clinicians with accessible decision support. This platform facilitates personalized assessment of individual AF recurrence risk and enables tailoring of therapeutic strategies accordingly.

In our study, the 1-year recurrence rate following RFCA in patients with NVAF was 36.00%, which is consistent with prior reports [14, 15]. Multivariate logistic regression analysis identified LAVI and RAVI related to atrial morphology and led to AF recurrence, similar to Takagi and Sallach’s research [16, 17]. A meta-analysis by Njoku et al. demonstrated that for every 1 mL/m^2^ increase in LAVI, the risk of AF recurrence rose by 3.20% [18]. RAVI > 30 mL/m^2^ was independently associated with an increased risk of composite clinical endpoint events in NVAF patients [19]. Research by Hopman et al. revealed that both left and right atrial underwent remodeling and myocardial fibrosis in AF patients, with strong interdependence between these processes [20].

Laboratory predictors of recurrence after RFCA in this study, consistent with prior reports [21–23], included SII, which comprehensively reflected the state of inflammation and immune imbalance [24]. In the current study, SII emerged as an independent predictor of recurrence after RFCA in NVAF patients, the elevated SII levels similar to the studies [25, 26], which indicated the inflammation might cause leukocyte infiltration, oxidative damage and atrial fibrosis, changes of atrial structure and electrophysiology, increasing the risk of recurrence [27–29].

In addition, patients with NYHA class III-IV had a significantly higher postoperative AF recurrence rate compared to those with NYHA class I-II. A prospective multicenter study by Sultan et al. [14] demonstrated significant differences in AF recurrence by NYHA status (40.00% recurrence rate vs 29.60% non-recurrence rate (*p* = 0.011), confirming NYHA classification as clinically relevant to AF recurrence. The pathophysiological basis was AF and heart failure exhibit bidirectional pathophysiological interactions. Heart failure may negatively affect left ventricular function, thereby indirectly impairing ablation outcomes [30].

Although the CHA₂DS₂-VASc score was identified as an independent predictor, the optimal cutoff value of 4.50 for predicting post-ablation recurrence in our cohort differed from previous reports [31]. The diagnostic performance analysis of Chao et al. [31] (sensitivity: 85.20%, specificity: 31.10%, AUC: 0.669) could effectively identify most patients with likely recurrence, but this cutoff value yielded substantial false-positive results. This discrepancy may stem from heterogeneous AF recurrence patterns across patient populations. When applied to broader cohorts, the cutoff may misclassify some low-risk patients (who would not recur) as high-risk and increases false-positive rates.

The nomogram had good discriminatory ability in the training and validation cohorts. A certain degree of validity and applicability of the model had been demonstrated, making our risk prediction more clinically attractive. This study provided further evidence in line with previous reports [31, 32], demonstrating that the goodness of fit of the model. The calibration curve showed that the calibration points of the training and validation cohorts were closely distributed along the 45 degree reference line, indicating that the predicted risk of the model was highly consistent with the actual results, which further confirmed its reliability.

Our DCA showed that the model provided a net benefit across a very wide threshold probability range of 20% to 60%. While this indicates robust performance, we have refined our interpretation to translate our model into clinical practice, we propose a risk-stratified management pathway based on the nomogram-predicted probability of recurrence. (1) Low-Risk ( < 20%): patients in this stratum may be managed with standard follow-up, including routine clinic visits and periodic ECG/Holter monitoring as per institutional protocol. The early discontinuation of antiarrhythmic drug (AAD) could be considered in this group. (2) Intermediate-Risk (20%-60%): for these patients, a more vigilant approach is warranted. This could include prolonged AAD therapy (e.g., for 6–12 months) and earlier or more intensive rhythm monitoring, such as repeated 24-hour Holter monitors or event monitors at 3 and 6 months to capture asymptomatic recurrences. (3) High-Risk ( > 60%): patients identified as high-risk should be counseled accordingly regarding their elevated likelihood of recurrence. Management should prioritize aggressive risk factor modification and close monitoring. This group may be considered for longer duration of antiarrhythmic drug (AAD) therapy post-ablation, earlier scheduled extended monitoring (e.g., with an implantable loop recorder) and should be prime candidates for prompt re-evaluation and discussion of a repeat ablation procedure should recurrence occur. However, in this analysis, the model employed a visual nomogram scoring tool to intuitively present prediction results. This approach not only significantly simplified physicians’ interpretation of recurrence risk assessments, but also enhanced precision in clinical decision-making, meanwhile improved workflow efficiency in clinical practice, finally provides critical support for personalized treatment strategies [33].

However, there are several limitations in this study. (1) As a single-center retrospective cohort study with a limited sample size, this work carries an inherent risk of overfitting the model. External validation using multi-center, prospective data from diverse populations is required to confirm its generalizability. (2) The use of stepwise selection for variable reduction is a recognized limitation. However, the clinical grounding of our candidate variables and the consistency of our key findings in sensitivity analyses support the validity of the presented model. Future studies with larger cohorts would benefit from applying penalized regression techniques (e.g., LASSO) for independent validation and exploration. (3) Using preoperative static data to establish the model, this study further investigates whether a different cut-off value might better stratify patients. (4) Surveillance for AF recurrence in our study relied on routine clinic ECGs and scheduled 24-hour Holter monitoring. This approach is inherently limited in its ability to detect asymptomatic or brief AF episodes. As a result, our study likely underestimates the true incidence of AF recurrence, leading to outcome misclassification that would generally bias the results toward the null. Therefore, the actual predictive performance of our nomogram, particularly its sensitivity, may be stronger than reported, had continuous long-term monitoring been used. Future studies utilizing implantable loop recorders or extended external monitoring could provide more complete arrhythmia detection and would be valuable for validating and potentially refining our model. (5) Our study did not incorporate electrophysiological parameters, such as left atrial voltage maps or the quantification of low-voltage areas, which are well-established markers of atrial substrate and fibrosis associated with recurrence risk. These parameters are typically acquired during invasive electrophysiology studies and were not routinely available for all patients in our cohort. (6) The nomogram incorporates five variables, including the NYHA class, CHA₂DS₂-VASc score, and SII index, each of which is a composite measure. The use of such indices carries the risk of oversimplification and multicollinearity. However, the prediction model developed in this study has performed well in feasibility, prediction efficiency, calibration and clinical practicability, which can provide important tool support for the risk assessment of postoperative recurrence in patients with NVAF. Future research should focus on integrating such pre-procedural clinical models with intra-procedural electrophysiological data to create comprehensive, multi-stage predictive algorithms that could further refine post-ablation management.

## Conclusions

In summary, this study developed a nomogram based on LAVI, RAVI, SII, NYHA functional classification, CHA₂DS₂-VASc score to estimate the risk of AF recurrence after RFCA. The newly developed nomogram demonstrated good discrimination and accuracy, suggesting its potential utility in predicting AF recurrence. Given its performance, the model is a promising tool; however, it is still in its preliminary stages and requires further validation with larger, multi-center, prospective studies involving diverse populations to confirm its generalizability.

## Electronic supplementary material

Below is the link to the electronic supplementary material.

Supplementary Material 1

Supplementary Material 2

## Data Availability

The authors confirm that the data supporting the findings of this study are available within the article.
