# Supplementary material for: Development and validation of a risk nomogram for predicting recurrence in patients with non-valvular atrial fibrillation after radiofrequency catheter ablation
Source: BMC Med Inform Decis Mak. 2026 Jan 10;26:22. doi: 10.1186/s12911-025-03338-4 (PMC12829022; doi:10.1186/s12911-025-03338-4)
Supplement: Supplementary file 1 — Supplementary Material 1 [file 12911_2025_3338_MOESM1_ESM.docx]

Table 1 Baseline Characteristics of Patients Excluded Due to Missing Data (N=21)

| Variables | Total  (N=21) |
| --- | --- |
| Age, **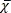**±s, y | 70.52±6.87 |
| Gender, n (%) |  |
| Male | 14 (66.67) |
| Female | 7 (33.33) |
| CHA_2_DS_2_-VASc Score,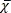±*s* | 3.33±1.24 |
| Type of AF, n (%) |  |
| Paroxysmal AF | 13 (61.90) |
| Non- paroxysmal AF | 8 (38.10) |
| Hypertension, n (%) |  |
| Yes | 18 (85.71) |
| No | 3 (14.29) |
| Diabetes, n (%) |  |
| Yes | 4 (19.05) |
| No | 17 (80.95) |
| NYHA classification, n (%) |  |
| Class I-Ⅱ | 8 (38.10) |
| Class Ⅲ-Ⅳ | 13 (61.90) |
| Echocardiographic parameters |  |
| LAVI, ml/m^2^ | 37 ± 12 |
| RAVI, ml/m^2^ | 23 ± 7 |
| LVDD, mm | 50.19±3.77 |
| LVDS, mm | 33.00±3.46 |
| LAD, mm | 44.68±8.97 |
| LVEF, % | 59.82±6.59 |
| TRSP, mmHg | 32.30 ± 8.50 |
| Laboratory values |  |
| eGFR, mL/min/1.73 m² | 44.68±8.97 |

AF: atrial fibrillation; NYHA: New York Heart Association; LAVI: left atrial volume index; RAVI: right atrial volume index; LVDD: left ventricular diastolic diameter; LVDS: left ventricular systolic diameter; LAD: left atrial diameter; LVEF: left ventricular ejection fraction; TRSP: tricuspid regurgitation systolic pressure; eGFR: estimated glomerular filtration rate.
