# Supplementary figures and images for: Development and validation of a risk nomogram for predicting recurrence in patients with non-valvular atrial fibrillation after radiofrequency catheter ablation
Source: BMC Med Inform Decis Mak. 2026 Jan 10;26:22. doi: 10.1186/s12911-025-03338-4 (PMC12829022; doi:10.1186/s12911-025-03338-4)

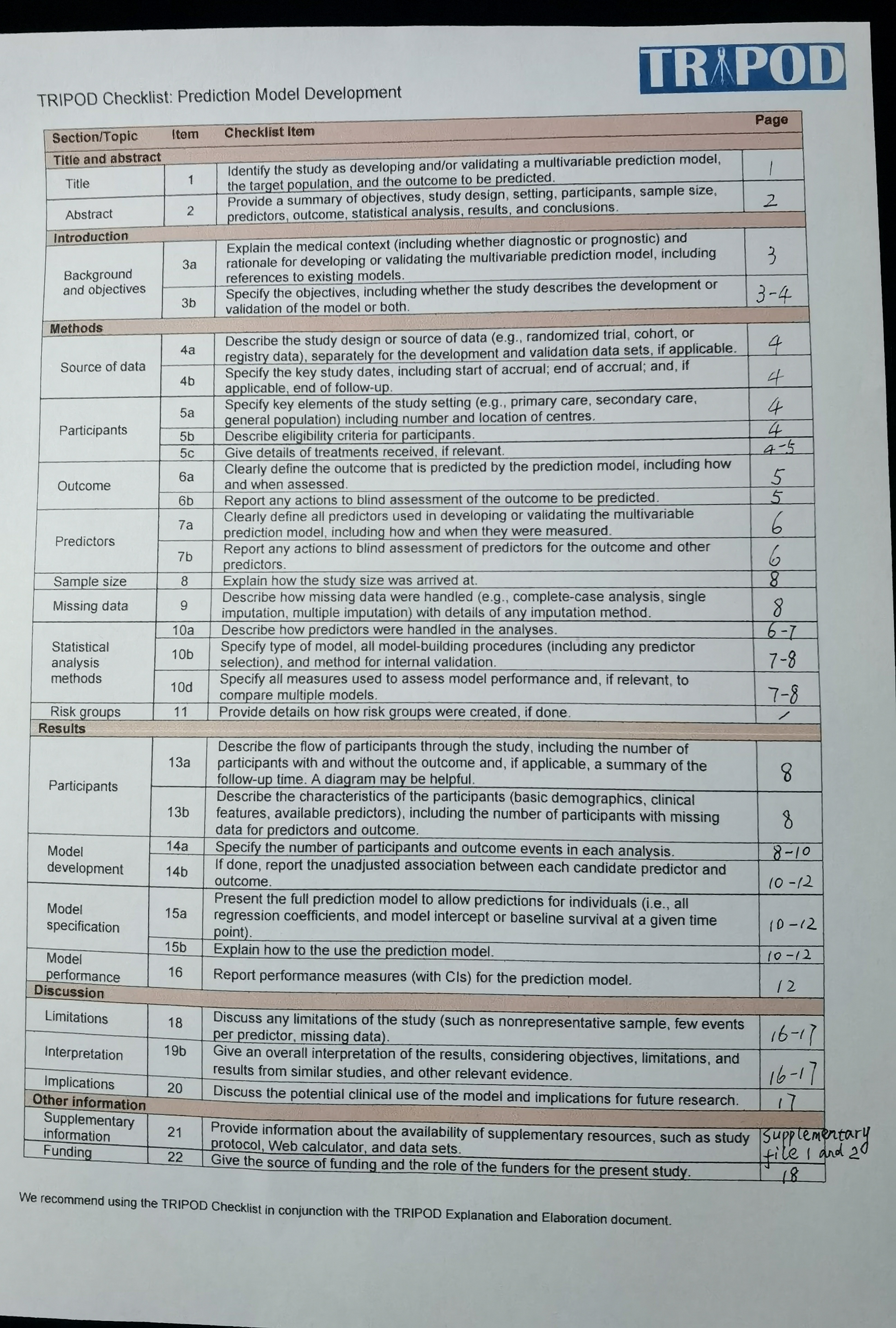

Supplement: Supplementary file 2 — Supplementary Material 2 [file 12911_2025_3338_MOESM2_ESM.jpg]
